# Identification of a novel heterozygous *PTH1R* variant in a Chinese family with incomplete penetrance

**DOI:** 10.1002/mgg3.2301

**Published:** 2023-10-16

**Authors:** Jie Wang, Chaoyue Zhao, Xin Zhang, Li Yang, Yanyan Hu

**Affiliations:** ^1^ Department of Pediatrics, Linyi People's Hospital Postgrad Training Base Jinzhou Med University Linyi China; ^2^ Department of Pediatrics Linyi People's Hospital Linyi China

**Keywords:** abnormal tooth eruption, incomplete penetrance, *PTH1R*, skeletal dysplasia

## Abstract

**Background:**

Mutations in *PTH1R* are associated with Jansen‐type metaphyseal chondrodysplasia (JMC), Blomstrand osteochondrodysplasia (BOCD), Eiken syndrome, enchondroma, and primary failure of tooth eruption (PFE). Inheritance of the *PTH1R* gene can be either autosomal dominant or autosomal recessive, indicating the complexity of the gene. Our objective was to identify the phenotypic differences in members of a family with a novel *PTH1R* mutation.

**Methods:**

The proband was a 13‐year, 6‐month‐old girl presenting with short stature, abnormal tooth eruption, skeletal dysplasia, and midface hypoplasia. The brother and father of the proband presented with short stature and abnormal tooth eruption. High‐throughput sequencing was performed on the proband, and the variant was confirmed in the proband and other family members by Sanger sequencing. Amino acid sequence alignment was performed using ClustalX software. Three‐dimensional structures were analyzed and displayed using the I‐TASSER website and PyMOL software.

**Results:**

High‐throughput genome sequencing and Sanger sequencing validation showed that the proband, her father, and her brother all carried the *PTH1R* (NM_000316) c.1393G>A (p.E465K) mutation. The c.1393G>A (p.E465K) mutation was novel, as it has not been reported in the literature database. According to the American College of Medical Genetics and Genomics (ACMG) guidelines, the p.E465K variant was considered to have uncertain significance. Biological information analysis demonstrated that this identified variant was highly conserved and highly likely pathogenic.

**Conclusions:**

We identified a novel heterozygous mutation in the *PTH1R* gene leading to clinical manifestations with incomplete penetrance that expands the spectrum of known *PTH1R* mutations.

## Graphical abstract

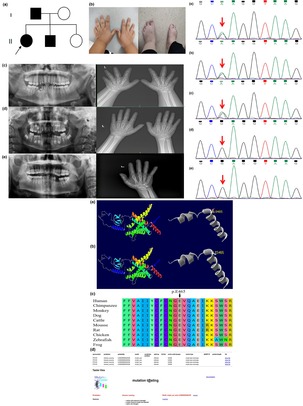

## 1 INTRODUCTION

Jansen‐type metaphyseal chondrodysplasia (JMC) is a rare skeletal dysplasia characterized by short‐limb dwarfism, hypercalcemia, hypercalciuria, and hypophosphatemia (Onuchic et al., 2012). Blomstrand osteochondrodysplasia (BOCD) presents with premature ossification of bones and can be grouped into two different types. Type I is a classic and severe form, characterized by skeletal malformation and shortened long bones, and most fetuses with this abnormality die in utero. Individuals with type II can exhibit mild manifestations (Oostra et al., 2000). Eiken syndrome is a skeletal dysplasia characterized by severely retarded ossification and abnormal bone remodeling. Recessive mutations in *PTH1R* have been identified in patients with Eiken syndrome and BOCD, but the clinical manifestations were opposite and might have different molecular underpinnings (Duchatelet et al., 2005). Enchondroma is characterized by abnormal proliferation and differentiation of chondrocytes. The tumor initially undergoes endochondral ossification near the growth plate cartilage and then gradually migrates toward the diaphysis (Couvineau et al., 2008). *PTH1R* pathogenic variants are found in 72% of primary failure of tooth eruption (PFE) cases with five clinical and radiographic phenotypes: involvement of posterior teeth, involvement of the distal teeth to the most affected mesial teeth, supraclavicular manifestations, altered vertical growth of the alveolar process and posterior open bite (Grippaudo et al., 2021). In this article, we identified a novel missense mutation in *PTH1R* with differential phenotypes in tooth development, facial features, and skeletal dysplasia. In addition, we summarized the incomplete penetrance of *PTH1R* mutations with a review of the literature. It is important that clinicians have a better understanding of the etiology, mechanism, and management of this condition.

## 2 MATERIALS AND METHODS

### 2.1 Ethical compliance

The study approved the examination of the ethics committee of Linyi People's Hospital (no.13003). The parents signed the informed consent form for clinical research.

### 2.2 Study subjects

**FIGURE 1:**
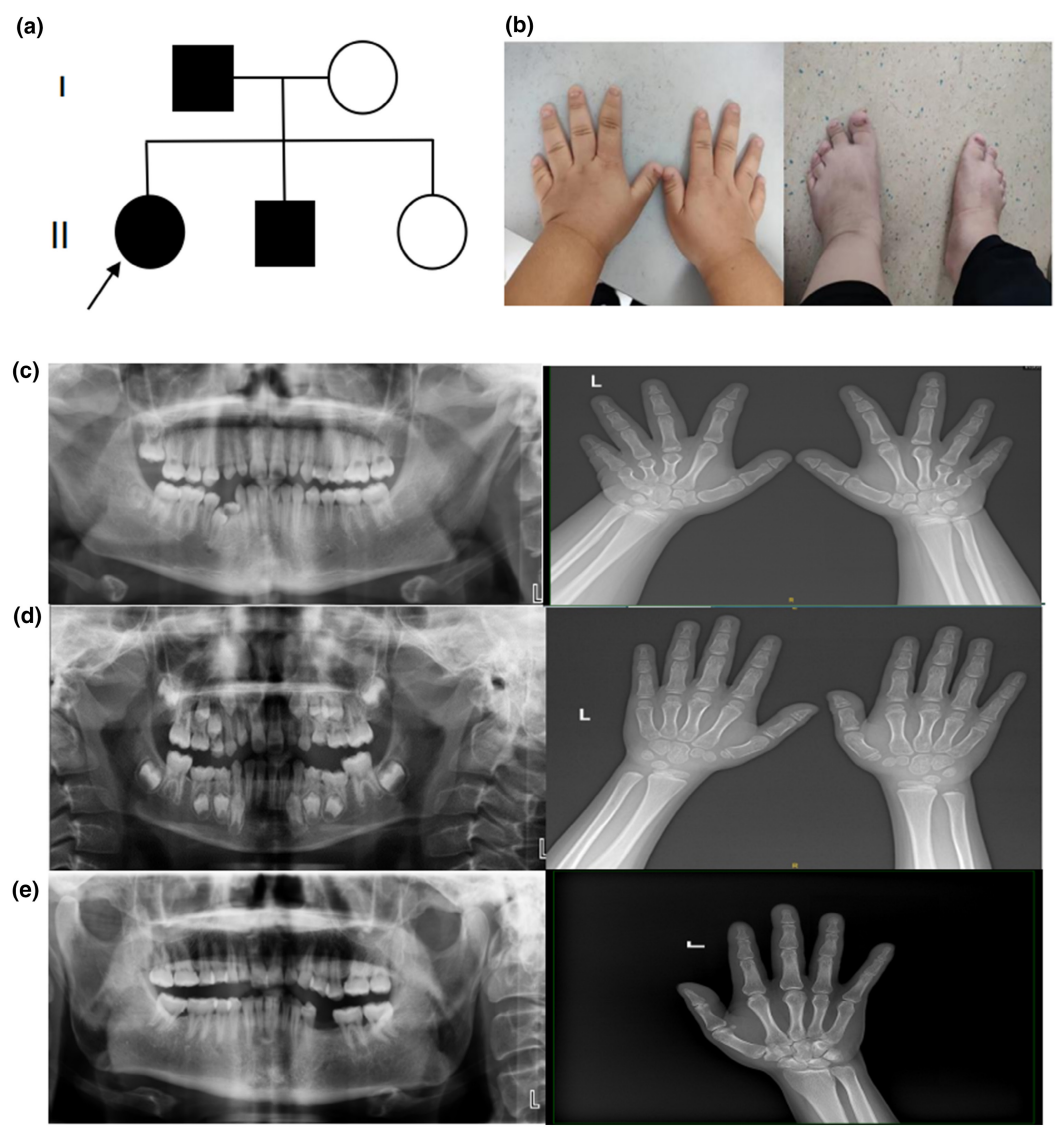
(a) Family tree: An arrow indicates the proband, filled symbols indicate affected individuals, circles denote females, and squares denote males. (b) Limb features of the proband. Oral and hand radiographs of individuals carrying heterozygous *PTH1R* variants: the proband (c), her brother (d), and her father (e).

The family (Figure 1a) comprised of five members. The proband, a 13‐year 6‐month‐old girl, was identified as having short stature and was referred to the hospital for investigation of the cause. She underwent surgery for hydrocephalus at 8 months of age. Developmental delay persisted throughout childhood. Physical examination showed a height of 138 cm (−3.38 SDS), weight of 57.8 kg, and head circumference of 57 cm. Facial features included a prominent forehead, low and flat nasal bridge, short and invaginated nasal columella, unique “half‐moon” nostril shape, convex upper lip, and misaligned teeth. Additional features included trident hands, stubbiness at the distal end of the extremities (Figure 1b), and cocked back of hips. Auxiliary test results were as follows (reference range is shown in brackets): serum parathyroid hormone (PTH), 64.02 pg/mL (10–69 pg/mL); calcium, 2.45 mmol/L (2.03–2.54 mmol/L); phosphorus, 1.48 mmol/L (0.9–1.34 mmol/L); magnesium, 0.82 mmol/L (0.7–1.1 mmol/L); alkaline phosphatase, 164.4 U/L (0–750 U/L); and 25‐hydroxyvitamin D, 22.24 ng/mL (≥20 ng/mL). The 24‐h urinary calcium excretion was 3.51 mmol/24 h (2.5–7.5 mmol/24 h), and the 24‐h urinary creatinine excretion was 10.82 mmol/24 h (7.1–17.7 mmol/24 h). Oral X‐rays revealed that the third molars on both sides of the maxilla and mandible had not erupted, and the mandibular right first premolar had not erupted due to deciduous teeth remaining in place. X‐ray of the hands showed that the third to fifth metacarpophalangeal bones were short (Figure 1c).

The brother of the proband was an 8‐year‐old boy with a height of 118 cm (−2.22 SDS), weight of 21.2 kg, and head circumference of 51.5 cm. He presented with misaligned teeth and blocked eruption of permanent teeth. Serum levels of phosphorus, calcium, 25‐hydroxyvitamin D, and PTH were normal. Oral X‐ray showed that some of the permanent teeth in the maxilla and mandible had not erupted; some of the deciduous teeth had not been removed, and some were misaligned. The X‐ray of the hands was normal (Figure 1d).

The father of the proband was a 38‐year‐old male with a height of 159 cm (−2.25 SDS), weight of 62.3 kg, and head circumference of 56.5 cm. He presented with short stature and teeth extraction after caries caused by incomplete eruption of permanent teeth 5 years ago. Serum levels of calcium, phosphorus, 25‐hydroxyvitamin D, and PTH were normal. Oral X‐ray revealed that wisdom teeth were missing in the maxilla and mandible, and the mandibular left second premolar was missing. The X‐ray of the hands was normal (Figure 1e).

### 2.3 Mutation screening and bioinformatics analysis

Peripheral blood samples (2 mL) were collected in EDTA anticoagulated venous blood tubes from all subjects. Libraries were constructed using an S220 Focused‐ultrasonicator (Covaris) and DNA Sample Prep Reagent Set and sequenced on DNBSEQ‐T7 with 150 bp paired‐end reads. Target‐capture next‐generation sequencing (NGS) was performed with genomic DNA using an 897‐gene panel, and the sequencing depth was 300x. The enrichment libraries were sequenced on an Illumina HiSeq X Ten sequencer with paired‐end reads of 150 bp. To obtain clean reads, raw reads in FASTQ format were filtered by removing adapters and low‐quality reads with cutadaptor software and Sentieon software. The SNP and INDEL variants were evaluated using Sentieon software, and annotated results were saved in VCF format. Potential deleterious effects were evaluated using SIFT, PolyPhen‐2, MutationTaster, and GERP++. ClustalX software was used to analyze amino acid sequence conservation among different species. Sanger sequencing was used to validate the variant in both the proband and other family members. The I‐TASSER model predicted the three‐dimensional structure of the protein, which was visualized using the PyMOL molecular graphics and modeling system.

In this study, four steps were used to select the potential pathogenic mutations in downstream analysis: (i) mutation reads were more than 5, and the mutation ratio was no less than 30%; (ii) The mutations should be removed, when the frequency of mutation was more than 5% in 1000 Genomes, ESP6500, and Inhouse database; (iii) the mutations should be dropped, if they were in InNormal database; (iv) the synonymous mutations were removed when they were not in the HGMD database. After that, the rest mutations should be the potential pathogenic mutations for further analysis.

## 3 RESULTS

### 3.1 Results of genetic testing and biochemical information analysis

**FIGURE 2:**
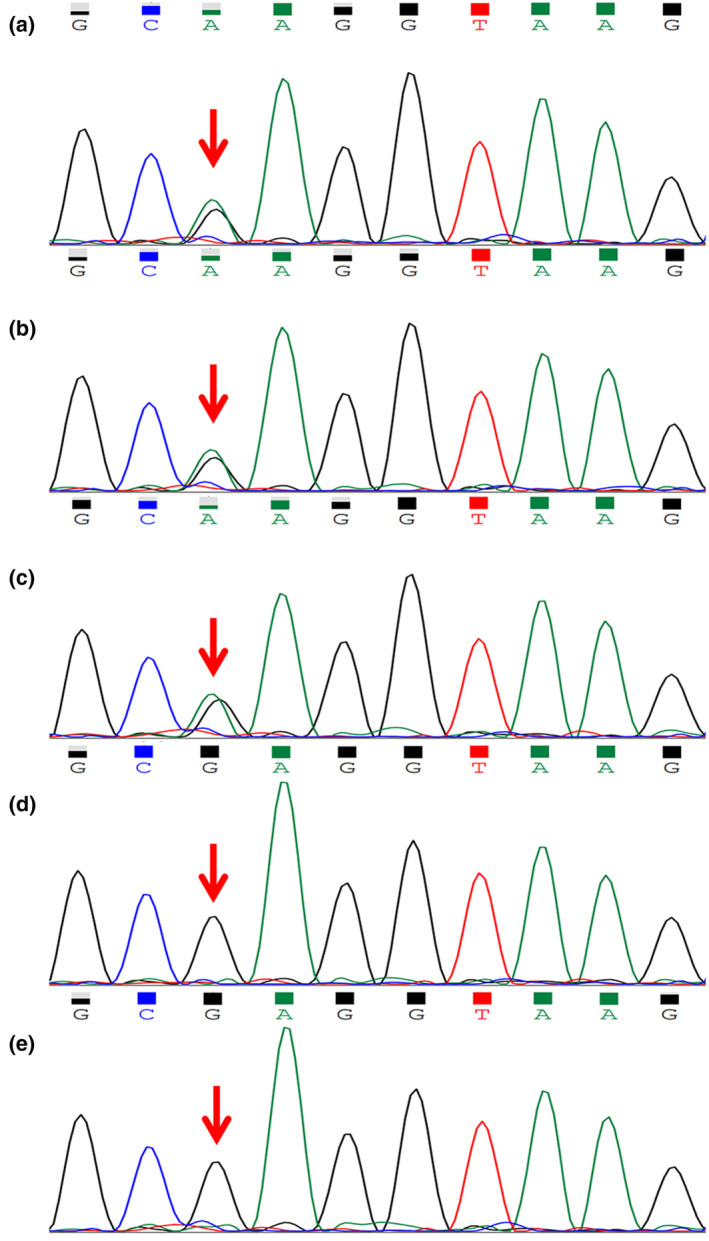
Sanger sequencing was performed for the proband and other family members. The same missense variant c.1393G>A (red arrow) in *PTH1R* was identified in the proband (a) and her father and brother (b, c), while her mother and sister (d, e) did not carry this variant.

A heterozygous de novo missense variant in exon 15 of *PTH1R* (NM_000316), c.1393G>A (p.E465K), was identified in the proband. Sanger sequencing confirmed the same heterozygous variant in her father and brother but not in other family members (Figure 2). This mutation site had not been reported previously and was predicted to be harmful by bioinformatics software. According to the ACMG guidelines, this mutation was classified as “Uncertain Significance” (PM2_Supporting + PP3).

**FIGURE 3:**
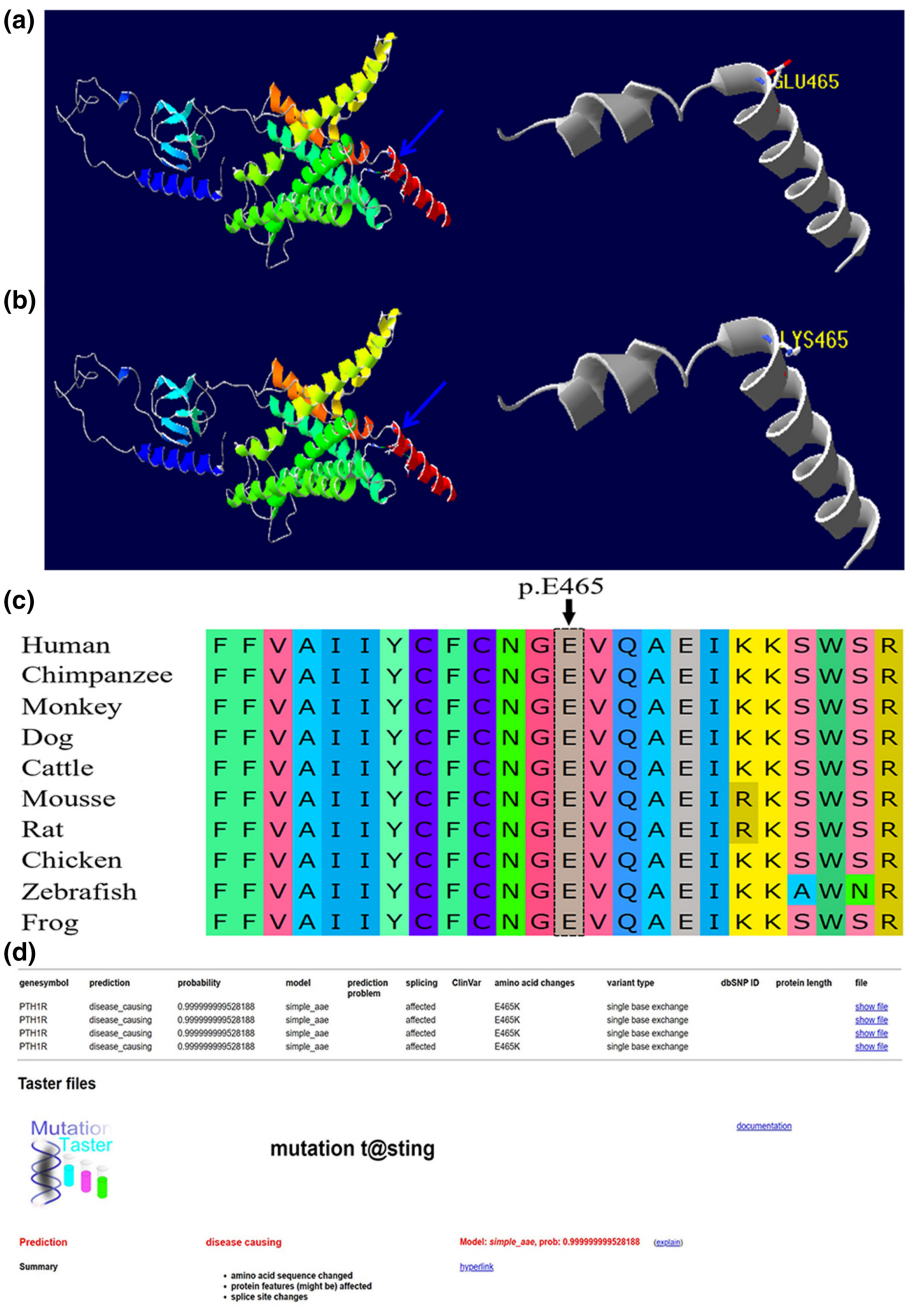
Three‐dimensional structure of the PTH1R protein and mutation locations (red box): (a) wild‐type PTH1R protein, (b) variant PTH1R protein. (c) Homologous comparison results showed that the amino acid residue of E465 is highly conserved among different species. (d) Mutation Taster revealed that this *PTH1R* mutation (c.1393G>A; p.E465K) was highly damaging (0.999).

The three‐dimensional structure of the *PTH1R* gene was generated by using I‐TASSER and displayed using PyMOL software (Figure 3a,b). Analysis of the amino acid sequence conservation of p.E465K in human, chimpanzee, monkey, dog, cattle, mouse, rat, chicken, frog, and zebrafish species indicated high conservation (Figure 3c). Mutation Taster (score = 0.9999) predicted that this variant was highly damaging (Figure 3d).

### 3.2 Literature review

To better understand the genotype–phenotype correlation, we reviewed the available literature using the PubMed and HGMD websites. Mutations in the *PTH1R* gene resulted in PFE, JCM, Eiken syndrome, BOCD, enchondroma, and pseudohypoparathyroidism. The most common mutations were missense mutations, and the most common disease was PFE. In addition, we collected the phenotypic differences of eight families with *PTH1R* mutations, which are summarized in Table 1. Our results suggested incomplete penetrance and variable expressivity of mutations in the *PTH1R* gene (Aziz et al., 2019; Bastepe et al., 2004; Beena et al., 2017; Decker et al., 2008; Frazier‐Bowers et al., 2010, 2014; Grippaudo et al., 2018, 2019; Guerreiro et al., 2016; Hopyan et al., 2002; Jacob et al., 2019; Jelani et al., 2016; Jobert et al., 1998; Kanno et al., 2017; Moirangthem et al., 2018; Nampoothiri et al., 2016; Onuchic et al., 2012; Rhoads et al., 2013; Risom et al., 2013; Roth et al., 2014; Savoldi et al., 2013; Schipani et al., 1996, 1999; Zhang et al., 1998). In our report, the proband exhibited a more severe abnormal tooth eruption and skeletal dysplasia phenotype than the other family members. To the best of our knowledge, this *PTH1R* mutation site has not been described previously. Furthermore, there was significant heterogeneity in disease severity even within families carrying the same gene mutation.

**TABLE 1:** Reports of different phenotypes in families with *PTH1R* gene variants.

Related diseases	Phenotype	PMID
BOCD	The siblings presented with facial deformities and skeletal dysplasia, nipple, and breast hypoplasia, but the elder brother presented with more severe phenotypes.	27353973
Enchondroma	The proband was affected by skeletal dysplasia and his father presented with mild clinical features.	11850620
JMC	This family presented with apparent abnormalities of skeletal development, but there were differences in laboratory tests. The mother's serum calcium, phosphorus, and PTH levels were within the normal range, while her two sons showed hypercalcemia, and decreased PTH levels.	27410178
	The onset age of the two brothers was different, and they presented with short stature, their father's height was P3. All of them presented with different forms of skeletal dysplasia.	15240651
Eiken syndrome	The family had normal biochemical results. However, one of them had a slight increase in serum PTH and developed type I diabetes at the age of 9 years.	15240651
PFE	Phenotype severity varies widely and more severe clinical manifestations in adults.	28257744
	The proband was diagnosed with PFE, and his mother had the same mutation site but had no PFE‐related phenotype.	31730001
	The proband, her cousin, and the cousin's mother all presented with an abnormal eruption of teeth, and one had osteoarthritis, while the proband's parents and maternal grandmother presented with a normal eruption of teeth.	24300310

## 4 DISCUSSION

*PTH1R* is a G protein‐coupled receptor with two distinct ligands, which that is the primary functional receptor for both the endogenous PTH and PTH‐related protein (PTHrP) ligands (Yamaguchi et al., 2011). PTHrP affects cartilage cell differentiation and bone remodeling. PTH promotes osteoblast differentiation and regulates calcium and phosphorus metabolism and mineral homeostasis (Martin et al., 2021). The *PTH1R* gene plays a key role in phosphorus and calcium metabolism and thus affects the state of bone tissue. It can stimulate osteoblast generation, and increase bone mass in both trabecular and cortical bone (Santa Maria et al., 2016).

The proband in this study presented with PFE, skeletal dysplasia, short stature, and midfacial hypoplasia, and she underwent hydrocephalus surgery at 8 months of age. Subramanian et al. (2016) suggested that signal transduction impaired by *PTH1R* variants occurs in the cells of periodontal tissues, leading to primary failure of tooth eruption. As shown in animal and human studies, PTHrP is expressed in the tooth germ and regulates the expression functional factors in periodontal tissue cells and osteoclast differentiation, leading to physiological root resorption of deciduous teeth and failure of permanent tooth eruption (Fukushima et al., 2005). In addition, mutations in *PTH1R* cause skeletal dysplasia and severe growth retardation. There are currently 436 descriptions of skeletal dysplasia syndromes, and skeletal dysplasia syndromes are the most common cause of short stature skeletal dysplasia (Olney & Bober, 2018). Bone lengthening is caused by an increase in chondrocyte number, synthesis of chondrocytes matrix, and substantial enlargement of chondrocyte. Proliferating chondrocyte columns are much shorter or even absent in mice lacking both copies of the PTH/PTHrP receptor, which delays chondrocyte differentiation (Kronenberg, 2006). Mice lacking *PTH1R* in osteoblasts have less trabecular bone formation, endochondral angiogenesis and vascular invasion of the cartilage are impaired, and hypertrophic development of chondrocytes is attenuated. A decrease in the number of hypertrophic chondrocytes, results in premature fusion of the growth plate and shortened long bones, with resultant short limbs (Qiu et al., 2015). Defects in endochondral bone formation, and the skull base and midface are affected, resulting in midface hypoplasia, anatomical abnormalities of the eustachian tube, and stenosis of the foramen magnum, with obstruction of cerebral circulation leading to hydrocephalus (Olney & Bober, 2018). In addition, all the patients in this family had normal serum calcium levels. Some scholars have proposed that different mutations may have pleiotropic effects on receptor function, including gain‐of‐function and loss‐of‐function effects. PTH‐mediated calcium homeostasis has mutagenic loss‐of‐function effects on calcium regulation in osteocytes and kidneys, and PTH normalizes serum calcium levels (Portales‐Castillo et al., 2023).

Analysis of the clinical data of eight families with *PTH1R* gene variants revealed that there were phenotypic differences in PFE, skeletal development, and biochemical characteristics. In our study, the proband's clinical phenotype and imaging test results were more severe than those of other affected family members, indicating that the variant has incomplete penetrance. Researchers have proposed the following four mechanisms involving the factors that affect the expression of penetrance: the severity of the genetic defect, genetic modifier factors, environmental impact, and mosaicism of alleles causing disease (Gruber & Bogunovic, 2020). In the *Caenorhabditis elegans* model, Raj et al. (2010) found that incomplete penetrance is intrinsically caused by random fluctuations in gene expression. Whether the pathogenic mutation is a missense, nonsense, or frameshift mutation does not significantly affect penetrance expression, and haploid insufficiency of the pathogenic gene causes incomplete penetrance (Kuehn et al., 2014). *PTH1R‐*mutant mice do not exhibit overt defects in tooth eruption (Ono et al., 2016), indicating that *PTH1R* haploinsufficiency is not sufficient to induce PFE (Yamaguchi et al., 2022). Epigenetic signals have significant effects on phenotypes. Ackermann (2015) observed that in the absence of genetic or environmental differences, phenotypic differences are largely due to gene expression and random assignment at cell division, another mechanism is epigenetic modification of chromatin by DNA methylation and/or changes in the composition of histones. A study of familial Mediterranean fever syndrome found that the phenotype of the disease was mild after migration from the Mediterranean to Europe, which may relate to differences in environmental factors, such as diet and infection (Ozen et al., 2014). Mensa‐Vilaró et al. (2019) found that individuals with gene mosaicism had a mild phenotype, whereas those without mosaicism had a pronounced phenotype, indicating that disease onset, progression, or severity is usually not apparent in the presence of gene mosaicism, possibly due to gene dosage.

It is also possible that the diverse clinical manifestations are the result of the incomplete penetrance of the *PTH1R* pathogenic variants. Hence, it is easy to ignore this gene as a cause of multiple diseases in clinical practice. Careful history taking, physical examination, and imaging examination should be performed for differential diagnosis. Finally, molecular genetic analysis should be performed to determine the underlying genetic cause.

## 5 CONCLUSIONS

In summary, early diagnosis, genetic counseling, and multidisciplinary follow‐up are essential for the treatment and prognosis of this disease. This study reports the difference in phenotypes in one family, and we found a new missense mutation at site 465 where glutamic acid was replaced by lysine (E465K), which enriches the pathogenic mutation spectrum of the *PTH1R* gene. Animal experiments are needed to validate the function of this gene and to further understand the potential molecular mechanism of these phenotypic differences.

## AUTHOR CONTRIBUTIONS

Jie Wang wrote the article. Chaoyue Zhao, Xin Zhang, and Li Yang collected the data. Yanyan Hu critically reviewed the article, and approved the final article as submitted. All authors approved the final version of the article.

## ETHICS STATEMENT

This study was approved by the Ethics Committee of Linyi People's Hospital.

## CONFLICT OF INTEREST STATEMENT

All authors declare that there is no conflict of interest.

## Data Availability

The data that support the findings of this study are available on request from the corresponding author. The data are not publicly available due to privacy or ethical restrictions.
